# Design of a multilayer-based collimated plane-grating monochromator for tender X-ray range

**DOI:** 10.1107/S1600577516017884

**Published:** 2017-01-01

**Authors:** Xiaowei Yang, Hongchang Wang, Matthew Hand, Kawal Sawhney, Burkhard Kaulich, Igor V. Kozhevnikov, Qiushi Huang, Zhanshan Wang

**Affiliations:** a MOE Key Laboratory of Advanced Micro-Structured Materials, Institute of Precision Optical Engineering, School of Physics Science and Engineering, Tongji University, Shanghai 200092, People’s Republic of China; b Diamond Light Source Ltd, Harwell Science and Inovation Campus, Didcot OX11 0DE, UK; c Shubnikov Institute of Crystallography of Federal Scientific Research Centre ‘Crystallography and Photonics’ of Russian Academy of Sciences, Moscow 119333, Russian Federation

**Keywords:** X-ray optics, monochromator, diffraction grating, multilayer

## Abstract

Collimated plane-grating monochromators (cPGMs), consisting of a plane mirror and plane diffraction grating, are essential optics in synchrotron radiation sources for their remarkable flexibility and good optical characteristics in the soft X-ray region. However, the poor energy transport efficiency of a conventional cPGM (single-layer-coated) degrades the source intensity and leaves reduced flux at the sample, especially for the tender X-ray range (1–4 keV) that covers a large number of *K*- and *L*-edges of medium-*Z* elements, and *M*-edges of high-*Z* elements. To overcome this limitation, the use of a multilayer-based cPGM is proposed, combining a multilayer-coated plane mirror with blazed multilayer gratings. With this combination, the effective efficiency of cPGMs can be increased by an order of magnitude compared with the conventional single-layer cPGMs. In addition, higher resolving power can be achieved with improved efficiency by increasing the blaze angle and working at higher diffraction order.

## Abstract 2

A multilayer-based collimated plane-grating monochromator (cPGM) with efficiency one order of magnitude higher than the traditional cPGM is proposed for the tender X-ray range. The resolving power can also be increased with improved photon flux by using a large blaze angle and working at high diffraction order.

## 1. Introduction   

**Figure 1:**
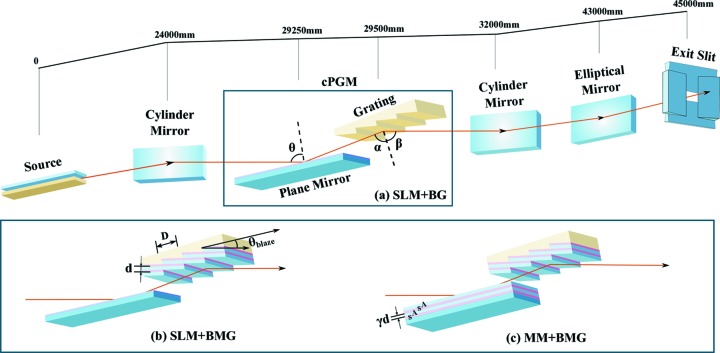
Schematic layout of Diamond I08 beamline. Different cPGM combinations are shown within the rectangle frame. (*a*) The conventional cPGM combination of a plane mirror and blazed grating both coated with a single metal layer (SLM+BG); (*b*) the single-layer grating is replaced with a blazed multilayer grating (SLM+BMG); (*c*) both the plane mirror and blazed grating (MM+BMG) are coated with multilayers.

Modern synchrotron radiation sources are capable of delivering X-ray beams with unprecedented coherence and brilliance although the ultimate beam performance depends on further development of optics employed in the beamlines. Collimated plane-grating monochromators (cPGMs) (Follath & Senf, 1997 ▸), composed of a plane mirror and plane diffraction grating each with a single-layer coating [Fig. 1(*a*)], are widely used to monochromatize soft X-ray (SXR) radiation from synchrotron sources due to their flexibility of switching among different modes for high flux, high spectral resolution or high harmonic suppression. However, the efficiency of the traditional grazing-incidence laminar gratings covered by a single reflecting coating is dramatically reduced, down to only a few percent above 2 keV, because of a sharp decrease (as 1/*E*) of the critical angle of total external reflection (TER) with increasing photon energy. An attempt to enhance the grating efficiency by replacement of the laminar grating with a blazed one was initiated by Cocco (Cocco *et al.*, 2007 ▸). Nevertheless, the measured efficiency still proved to be not more than 20% at photon energies exceeding 2 keV with the small critical angle of TER remaining as the primary limitation of efficiency. Meanwhile, crystal monochromators demand a complicated design for energies below 4 keV and suffer from heat problems below 2.5 keV.

To solve the problem of efficiency reduction in the tender X-ray range, for example 1–4 keV, multilayer grating structures have been proposed, produced by either depositing alternating layers of high- and low-*Z* materials onto a laminar or blazed grating surface or by etching a grating structure in the multilayer mirror. We can refer to one of the first successful efforts, namely the spherical multilayer coated grating designed for the SXR monochromator operating in this interval of the photon energies (McNulty *et al.*, 1997 ▸). During the last few years, so-called alternate multilayer gratings have been designed and installed in several synchrotron beamlines, reaching 27% efficiency at 2.2 keV (Choueikani *et al.*, 2014 ▸; Ohresser *et al.*, 2014 ▸; Vantelon *et al.*, 2016 ▸; Belkhou *et al.*, 2015 ▸). Recently, blazed multilayer gratings (BMGs) with 35% efficiency at 2 keV and a maximum efficiency of 55% at 4 keV have been developed at BESSY-II (Schäfers *et al.*, 2016 ▸; Senf *et al.*, 2016 ▸). In parallel, BMGs have also been intensively studied at the LBNL for high-resolution SXR spectroscopy where a record diffraction efficiency of 52% has been obtained at 13.4 nm (Voronov *et al.*, 2011 ▸, 2014 ▸).

To fully exploit the capability of BMGs we compare the performance of different BMG-based cPGMs. We have shown that the efficiency of the proposed multilayer-based cPGM can be improved by one order of magnitude compared with a conventional single-layer-based cPGM after 3 keV. Moreover, we also propose ways to increase the resolving power for the proposed cPGM with improved efficiency.

The following analysis is based on the Scanning X-ray Microscopy beamline I08 at Diamond Light Source (Diamond), and a schematic layout is presented in Fig. 1 ▸. The beamline operates in the energy range spanning from 0.25 keV up to 4.4 keV with photons being produced by a 4.5 m-long APPLE II undulator. At 3 keV the RMS horizontal source and divergence are 123 µm and 25 µrad, respectively, whereas along the vertical direction the values are 12 µm and 8 µrad. As shown in Fig. 1 ▸, the SXR radiation emitted by the undulator is first collimated in the vertical plane by the horizontally deflecting cylinder mirror and then dispersed vertically by the plane mirror and grating. After twofold reflection from a second vertically focusing cylinder mirror and a horizontally focusing elliptical mirror the stigmatic beam falls onto the exit slit. The distances between the optical elements are indicated in Fig. 1 ▸. The SXR beam falls onto the collimating and focusing mirrors at the same grazing angle of 0.7° allowing them to reflect radiation up to a photon energy of 4.4 keV. The current cPGM system has four grating substrate cartridges, which hold two laminar gratings for energies below 2 keV, one blazed grating for energies from 2 to 4.4 keV, and a spare substrate cartridge for future upgrade with a high-efficiency multilayer grating.

## 2. Comparison of different cPGM efficiency   

For definiteness, the following comparison between a single-layer cPGM and different BMG-based cPGMs is undertaken with a fixed grating line density of 600 lines mm^−1^ determined by the moderate resolving power requirement at I08 beamline. The coating material for the conventional blazed grating (BG) is gold (Au), and the blaze angle is set to 0.4° for total reflection.

The parameter choice is more complicated for a multilayer-based cPGM. The operating principle for a BMG is to simultaneously satisfy the grating equation and Bragg law for multilayers, and maximal efficiency occurs in the single-order regime when
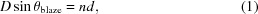
where θ_blaze_ is the blaze angle, *n* is the diffraction order, *d* is the multilayer period, and *D* is the grating period (Yang *et al.*, 2015 ▸). We assume that the periodic multilayer structure consists of two alternating materials, absorber A and spacer S, with the effects of interlayers and interfacial roughness being neglected. The Cr/C multilayer is selected because both chromium and carbon are free from absorption edges in the working spectral range and the fabrication technology of Cr/C multilayers has been well developed to date (Tu *et al.*, 2014 ▸; Wen *et al.*, 2015 ▸). The multilayer thickness ratio γ, *i.e.* the ratio of chromium layer thickness to the multilayer period, is set to 0.4, and the number of bi-layers is chosen to achieve the maximum possible reflectivity. For the purposes of illustration, two BMGs are analysed below. One is denoted as BMG_0.5_, having a 0.5° blaze angle and 14.54 nm multilayer period, and the other is denoted as BMG_0.4_, characterized by a 0.4° blaze angle and 11.64 nm multilayer period. Both BMGs work at the −1st order, the same operating order for conventional PGMs, and the multilayers coated on the plane mirror in this paper are the same as their corresponding BMG if not otherwise specified.

The focusing parameter 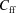 = 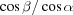, where α and β are the incident and diffraction angles to the grating normal as schematically shown in Fig. 1 ▸, is usually used to describe the working geometry and adjust the desired flux or resolving power of conventional blazed gratings. Blazed multilayer gratings achieve maximum efficiency at different incidence angles for different photon energies, while conventional blazed gratings rely on total reflection and can work at a range of incidence angles within the total reflection regime. Since the *C*_ff_ value of BMG_0.4_ at the central energy 3 keV is 1.7, we set the same value for conventional BGs for fair efficiency comparison.

**Figure 2:**
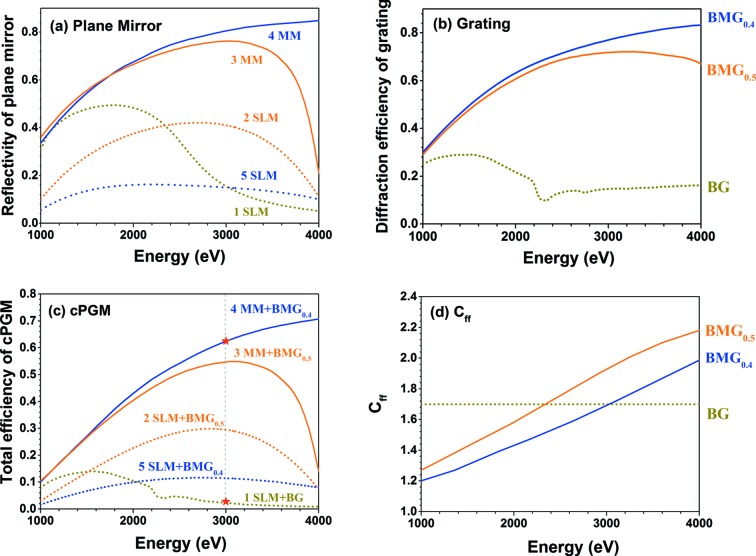
(*a*) Reflectivity of plane mirrors with single-layer (SLM) or multilayer (MM) coatings. (*b*) The −1st order diffraction efficiency of BG, BMG_0.5_ and BMG_0.4_. (*c*) The −1st order diffraction efficiency for different cPGM combinations. (*d*) Corresponding *C*_ff_ value. BMG_0.5_ is characterized by the following parameters: θ_blaze_ = 0.5°, *d* = 14.54 nm, 10 bi-layers. BMG_0.4_: θ_blaze_ = 0.4°, *d* = 11.64 nm, 20 bi-layers. BG: Au coating, θ_blaze_ = 0.4°, *C*_ff_ = 1.7. All SLMs are plane mirrors coated with a Ni layer having no roughness. The multilayer plane mirror (MM) placed in front of the BMG is coated with the same multilayer structure as the BMG. All gratings have 600 lines mm^−1^ line density. The dashed line in (*c*) signifies 3 keV and red stars denote the efficiency of curve 1 and 4 at 3 keV.

A comparison between cPGMs of different designs is presented in Fig. 2 ▸, where the reflectivity of plane mirrors with single and multilayer coatings, grating efficiencies and total cPGM efficiencies are shown. The corresponding *C*_ff_ values are given in Fig. 2(*d*) ▸ with the same colour. The simulations of the single-layer blazed gratings and plane mirrors are carried out with *REFLEC* software (Schafers & Krumrey, 1996 ▸), and the reflectivities of the multilayers are calculated using the *IMD* program (Windt, 1998 ▸). The calculations for BMGs are based on a rigorous coupled wave approach well suited for analysis of SXR multilayer gratings of various types with different shape of grating grooves (Kozhevnikov *et al.*, 2010 ▸; Yang *et al.*, 2015 ▸; van der Meer *et al.*, 2013 ▸). The simplest model of the BMG is considered in the present paper, assuming 90° anti-blaze angle and neglecting the effects of both interfacial roughness and corrugations of interfaces above the anti-blazed facets.

Firstly, the peak efficiencies of three gratings at different energies are calculated as shown in Fig. 2(*b*) ▸. The efficiencies of both BMGs are more than six times higher than that of the conventional blazed grating (BG) above 3 keV. The reflectivity of the plane mirror is determined by the characteristic of cPGMs that the outgoing beam should be parallel to the incoming one. This results in the following geometrical relation between the diffraction angle β and the incidence angles θ and α on the plane mirror and grating, respectively:
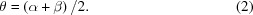
With the incidence and diffraction angles of the gratings and the geometrical relation given by equation (2), we can obtain Fig. 2(*a*) ▸ showing the reflectivities of the plane mirrors having a Ni single-layer (SLM) coating for all three gratings and a multilayer coating (MM) for the two multilayer gratings.

Fig. 2(*c*) ▸ is the product of the plane mirror reflectivities in Fig. 2(*a*) ▸ and the efficiency of the gratings in Fig. 2(*b*) ▸. The total efficiency of the conventional cPGM (curve 1) is increased from under 5% to over 25% when the single-layer Au blazed grating is replaced with Cr/C BMG_0.5_ [curve 2 in Fig. 2(*c*) ▸]. Higher efficiency, up to 50%, can be achieved by coating the same multilayer structure on the plane mirror in front of BMG_0.5_ (curve 3). The efficiency drop of BMG_0.5_ at high energy can be avoided by decreasing the multilayer period, for example to 11.64 nm (curve 4). The efficiency increases by one order of magnitude when compared with the conventional ones at 3 keV with the same *C*_ff_ value. Fig. 2(*d*) ▸ represents the *C*_ff_ value of each grating.

The efficiency drop of the BMG_0.5_-based cPGM after 3 keV is initially caused by BMG_0.5_ nearing the total external reflection region as shown in Fig. 2(*b*) ▸ by a slowly flattening curve. This leads to a shift of the efficiency peak of BMG_0.5_ at the edge of the total reflection region, and lowers the diffraction efficiency of the grating. More importantly, it deviates the incidence angle of the multilayer on the plane mirror from its position for peak reflectivity, reducing both the reflectivity of MM [curve 3 in Fig. 2(*a*) ▸] and the efficiency of the cPGM [curve 3 in Fig. 2(*c*) ▸].

When the blaze angle is much smaller than the total reflection angle, the refraction effect differences between the BMG and MM can be neglected. This means that the incidence and diffraction angle of the BMG approximately equals the Bragg angle of the multilayer plus or minus the blaze angle (Yang *et al.*, 2015 ▸), and equation (2) can be written as
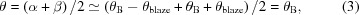
where θ_B_ is the Bragg angle of the multilayer. For a cPGM in the energy range 1–4 keV, a structure like BMG_0.4_ fulfils this condition and its corresponding cPGM has high efficiency. On the other hand, we can always shift the multilayer period on the plane mirror for the desired efficiency distribution of a cPGM.

Meanwhile, grating BMG_0.4_ has a smaller incidence angle α than the grating BMG_0.5_, which decreases the incidence angle θ of the plane mirror. Based on total reflection, the single-layer mirror for grating BMG_0.4_ would have a lower reflectivity [curve 5 in Fig. 2(*a*) ▸] and, consequently, low efficiency of their combination in a cPGM [curve 5 in Fig. 2(*c*) ▸]. Since gratings BMG_0.4_ and BMG_0.5_ have similar diffraction efficiency, the comparison between curves 1, 2 and 5 in Fig. 2(*c*) ▸ shows the importance of the plane mirror reflectivity. Ultimately, it is the combination of plane mirror and grating that decides the final efficiency.

## 3. Analysis of grating parameters influencing the resolving power   

Although the efficiency of the cPGM can be greatly improved by the use of multilayer coatings, a multilayer-based cPGM does not offer the benefit of providing a free choice of *C*_ff_ value, which is the parameter used in conventional cPGMs to modify resolving power.

The two parameters to which the resolving power of the grating is proportional are the diffraction order and the total number of grooves being illuminated on the surface. Single-layer blazed gratings rely on total reflection and the small total reflection angle in the tender X-ray region means that the grating can only work at the ±1st order for higher efficiency. At fixed diffraction order, we can change the incidence angle of the grating to alter the resolving power as denoted by the *C*_ff_ value. The larger the *C*_ff_ value, the smaller the grazing-incidence angle, and hence a greater number of grooves are illuminated which leads to an increase in resolving power.

Different from a single-layer grating, the multilayer grating cannot freely alter its *C*_ff_ value. The *C*_ff_ value, *i.e.* the incidence and diffraction angle, of the multilayer grating which maximizes its efficiency is determined by the BMG structure and varies with photon energy. Moreover, the grazing-incidence angle of the multilayer grating is larger than the total reflection angle which forces the BMG to use a smaller *C*_ff_ value (for example, *C*_ff_ = 1.7 at 3 keV for grating BMG_0.4_) than typically used with conventional blazed gratings to achieve high resolving power (*e.g.* *C*_ff_ = 4) and results in a lower resolving power.

This leads to the consideration of using higher diffraction orders to achieve higher resolving power. Since the multilayer grating does not rely on total reflection, it is not restricted to the ±1st order as for the single-layer grating. If the *C*_ff_ value of the multilayer grating remains the same or increases with diffraction order, the resolving power can be improved at higher diffraction order. However, the *C*_ff_ value decreases at larger diffraction order, which makes the resulting change of resolving power uncertain. At first glance, we can multiply the *C*_ff_ value and grating diffraction order to compare the resolving power, but, since the influence of these two parameters on resolving power are different, the product is inconsequential.

On the other hand, there are other ways to decrease the optimum grazing-incidence angle of the BMG in order to increase the number of grating grooves being illuminated: either increase the blaze angle or the multilayer period. In fact, the blaze angle and the multilayer period are coupled at fixed diffraction order and line density according to equation (1). Here we analyze the problem through the blaze angle parameter.

**Figure 3:**
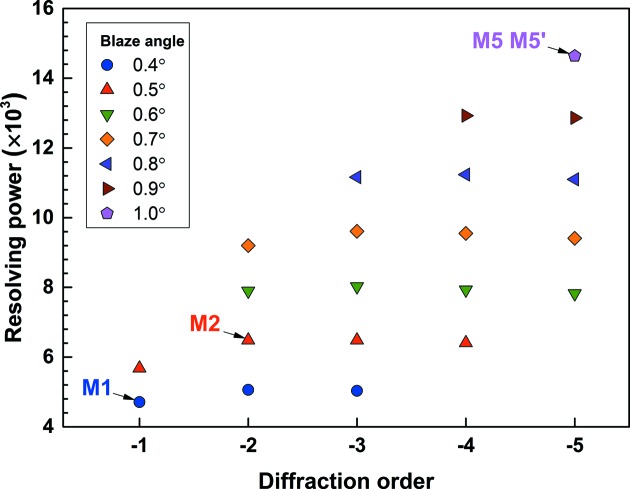
The influence of grating order and blaze angle on resolving power at 3 keV with the Cr/C BMG having 600 lines mm^−1^ line density. M1, M2, M5 and M5′ indicate the BMGs chosen for calculation in Fig. 4 ▸. M1: −1st order, θ_blaze_ = 0.4°, *d* = 11.64 nm; M2: −2nd order, θ_blaze_ = 0.5°, *d* = 7.27 nm; M5 and M5′: −5th order, θ_blaze_ = 1.0°, *d* = 5.82 nm.

In order to know how diffraction order and blaze angle influence the resolving power, we can write out a relation linking the three parameters. When the BMG structure is in the single-order regime and both incidence and diffraction angles are not close to the TER region, the general Bragg equation of the BMG can be written as
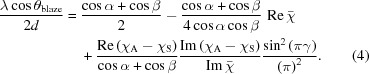
Here, 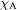 and 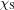 are the polarizability of absorber A and spacer S, respectively, and 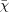 = 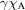 + 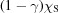 is the mean polarizability of the multilayer structure. Substituting the approximation 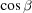 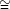 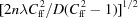, we can obtain the influence of the blaze angle and grating order on the *C*_ff_ value as follows,
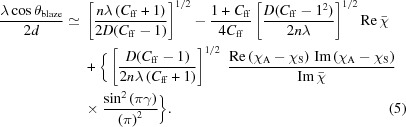
Considering the analytical resolving power expressions from Follath & Senf (1997 ▸) and equation (5), we can connect the blaze angle and grating order parameters with the resolving power of the BMG as illustrated in Fig. 3 ▸; this is also the resolving power of its corresponding cPGM. When the incidence or diffraction angle is close to the TER region, the accuracy of equation (5) decreases.

According to equation (1), the multilayer period increases with larger blaze angle and smaller diffraction order. The resolving power calculation includes the contribution of the finite source size, slope errors of the focusing mirror, plane mirror, grating and exit slit size. Realistic slope errors are used: the cylinder and elliptical mirror have been given tangential and sagittal r.m.s. slope errors of 1 µrad and 5 µrad, respectively. The tangential and sagittal r.m.s. slope errors have been set to 0.5 µrad for the plane mirror and 0.2 µrad for the grating. The vertical opening of the exit slit is set to 10 µm.

In Fig. 3 ▸, we calculate the resolving power of different BMGs with blaze angles ranging from 0.4° to 1.0° and grating order from −1st to −5th at 3 keV. Blaze angles are categorized by color. As anticipated, the resolving power at the same grating order increases with larger blaze angle. For the same blaze angle, the resolving power is not necessarily bigger at higher grating orders since the *C*_ff_ value decreases at higher diffraction order. However, because of the correlation between multilayer period, blaze angle and diffraction order, the symbols in Fig. 3 ▸ are not complete and larger blaze angle usually means higher diffraction order. The multilayer structure has a limited practical range of *d*-spacing due to the stress, roughness and crystallization problems during fabrication. For the Cr/C combination, the practical multilayer period range is approximately within 3–20 nm (Niibe *et al.*, 1992 ▸). Apart from this, a multilayer period that is too large would bring the incidence angle into the TER region, as the grazing-incidence angle of the BMG is approximately the Bragg angle minus the blaze angle. If the blaze angle exceeds the Bragg angle of the multilayer structure, there would be no diffraction wave coming out. This limitation further reduces the practical parameters to those still left in Fig. 3 ▸. This map shows that, for a BMG with a certain line density, we should use a large blaze angle for higher resolving power as long as the incidence angle is not close to the TER region, and we can use this map to find the blaze angle and diffraction order for the BMG to obtain the desired resolving power.

The concerns for multilayer-based cPGMs working at larger blaze angle and higher diffraction order is that the peak efficiency of the BMG decreases with larger blaze angle (Yang *et al.*, 2015 ▸), and that the accuracy of equation (3) becomes poor. Since the blaze angles that we considered here are relatively small, the reduction in BMG peak efficiency is not dramatic, while the poor approximation of equation (3) may be a real problem. The inaccuracy means that the incidence angle for the plane mirror deviates from the Bragg angle of the multilayer. The deviation angle is small, but the smaller the multiplayer period is, the more sensitive the reflectivity is due to its narrower bandwidth. Additionally, the requirement on the precision of the multilayer period becomes stricter and makes the achievement of high efficiency much more difficult.

For demonstration, we choose three BMGs from Fig. 3 ▸ for comparison: M2 designed for −2nd order with 0.5° blaze angle (*d* = 7.27 nm, 40 bi-layers), M5 and M5′ for −5th order with 1.0° blaze angle (*d* = 5.82 nm, 60 bi-layers), together with M1 designed for −1st order with 0.4° blaze angle (*d* = 11.64 nm, 20 bi-layers) as calculated in Fig. 2 ▸. For each BMG, the same multilayer is coated on the plane mirror except M5′ having slightly different multilayer period (*d* = 5.73 nm) on the plane mirror.

**Figure 4:**
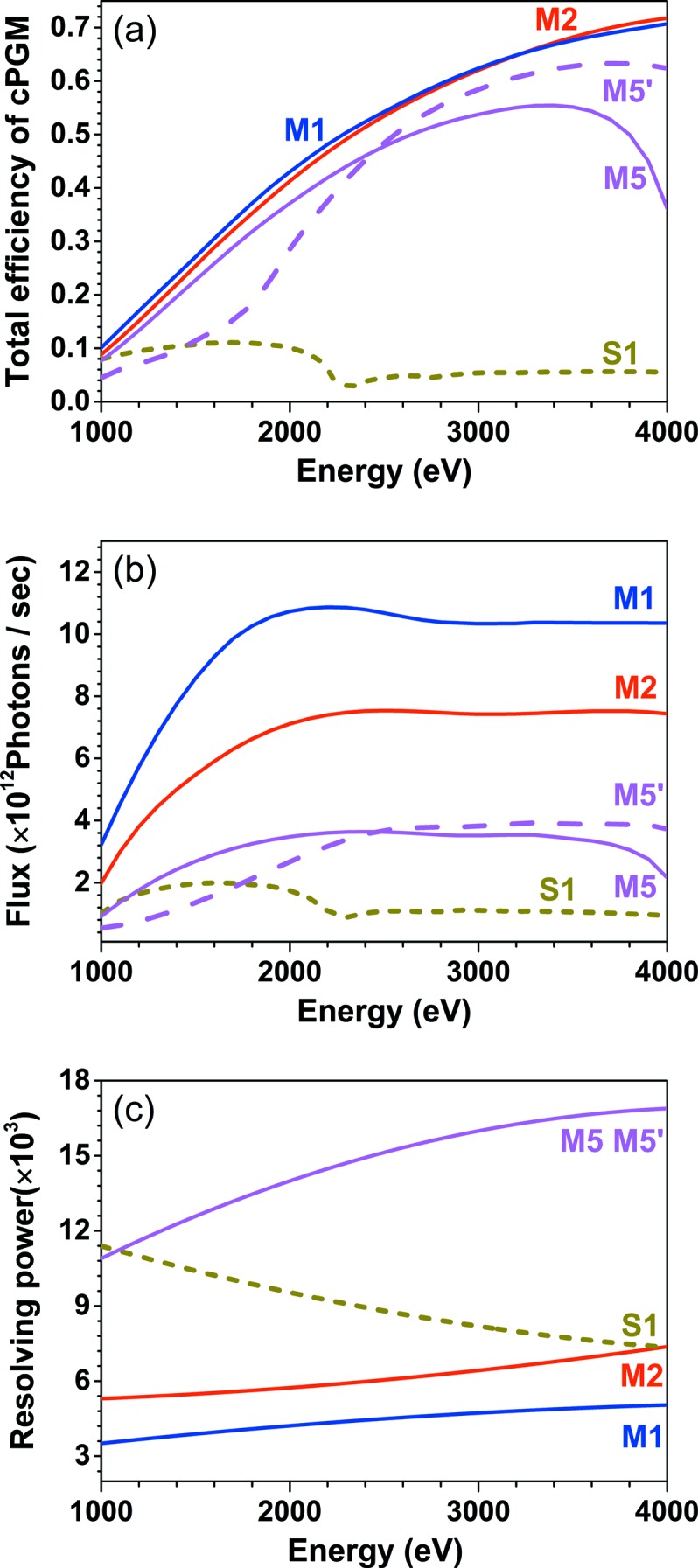
(*a*) Total efficiency of Cr/C multilayer-based cPGMs designed for different diffraction orders, (*b*) flux at sample and (*c*) their resolving powers. Detailed parameters of the I08 beamline used for flux and resolving power simulation in *SHADOW* are described in §1 and the same realistic figure errors are used as in Fig. 3 ▸. The slit size is 10 µm. The parameters of multilayer-based cPGMs are: M1: −1st order, θ_blaze_ = 0.4°, *d* = 11.64 nm, 20 bi-layers; M2: −2nd order, θ_blaze_ = 0.5°, *d* = 7.27 nm, 40 bi-layers; M5: −5th order, θ_blaze_ = 0.5°, *d* = 5.82 nm, 60 bi-layers; M5′: same BMG as M5 but *d* = 5.73 nm for multilayer coating on the plane mirror. The conventional cPGM is denoted as S1 having a nickel-coated plane mirror and gold-coated blazed grating with θ_blaze_ = 0.4° and *C*_ff_ = 4.

The total efficiencies of BMG-based cPGMs are presented in Fig. 4(*a*) ▸, while Fig. 4(*b*) ▸ illustrates the flux at the sample with each cPGM using the I08 beamline layout, and Fig. 4(*c*) ▸ reveals their corresponding resolving power in the same colour. Here, the resolving power and energy flux (photons s^−1^) at the sample position are taken from the result of ray tracing using *SHADOW* (Sanchez del Rio *et al.*, 2011 ▸). The efficiency, flux and resolving power of the conventional cPGM with single-layer coating on both the plane mirror and grating working at −1st order and high-resolution mode (*C*_ff_ = 4) are also shown in Fig. 4 ▸ as curve S1 for reference.

As shown in Fig. 4(*a*) ▸, the total efficiency of the BMG-based cPGM designed for −1st and −2nd order (M1, M2) is almost the same, while M5 designed for the −5th order with larger blaze angle and smaller multilayer period has lower efficiency. The efficiency drop comes from poor approximation in equation (3), *i.e.* bigger deviation in θ_B_ − θ_blaze_ and α, that leads to the incidence angle of the plane mirror deviating from the Bragg angle of the multilayer structure with the smaller multilayer period narrowing the reflectivity peak width and further enhancing the reduction. Higher efficiency after 2.5 keV can be obtained if we shift the multilayer peak position to accommodate the obtained incidence angle θ by decreasing the multilayer period on the plane mirror to, for example, 5.73 nm (curve M5′). Coating two stripes on the plane mirror can cover the target energy range in combination, but this increases the complexity of the system.

In Fig. 4(*c*) ▸ the resolving power of the cPGM is shown. As anticipated in Fig. 3 ▸, the resolving power continues to increase from M1 to M5. The lowest resolving power curve corresponds to the M1 designed for the −1st order and 0.4° blaze angle due to its small *C*_ff_ value. A 25% increase of resolving power at 3 keV can be obtained using M2 designed for the −2nd order and 0.5° blaze angle. The highest resolving power curve at 1–4 keV, even exceeding the typical value in high-resolving-power mode of conventional cPGMs, belongs to M5 or M5′ designed for the −5th order and 1.0° blaze angle. At 3 keV, the resolving power for M5 or M5′ is 1.6 × 10^4^, which is even higher than that (1.1 × 10^4^) using a Si(111) double-crystal monochromator.

It seems that the BMGs designed for larger blaze angles have both relatively high efficiencies and resolving power; however, the flux at the sample would be quite different as shown in Fig. 4(*b*) ▸. Given the same slit size, the BMG with high resolving power has lower flux passing through the slit. It is reasonable to compare the resolving power of two structures with the same flux or *vice versa*. From Fig. 4(*c*) ▸ we can see that the resolving power of conventional cPGM S1 coincides with the BMG-based cPGM M2 at 4 keV, while the latter has around eight times higher flux at the sample due to the higher efficiency of the multilayer. For comparison, the calculated flux for a double-crystal monochromator would be 4.8 × 10^12^ photons s^−1^, while that for M5 or M5′ would be 3.5 × 10^12^ photons s^−1^ at 3 keV. However, it should be noted that it becomes more challenging to use the double-crystal monochromator at lower energy since the grazing-incidence angle for a crystal is about 41° and 81° at 3 keV and 2 keV, respectively.

Based on the above discussion, we can conclude that a larger blaze angle contributes to a higher resolving power and a higher diffraction order is not necessarily related to a higher resolving power. Also, while limited by the practical multilayer period, a large blaze angle usually is linked with high diffraction order. The ±1st order may be the only choice for conventional cPGMs, but not the only choice for the multilayer-based cPGMs. Note that the plane mirror should always be coated with a multilayer for high-order cPGMs, since the grazing-incidence angle for the plane mirror would be larger and away from the TER region. In addition, we need to be aware that the angle deviation between the Bragg angle of the multilayer and the incidence angle θ obtained from the cPGM geometrical requirements increases for larger blaze angle, and the narrower peak bandwidth caused by the smaller multilayer period would add harsh requirements on the multilayer period fabrication precision and angle alignment.

If we consider a wideband BMG with a depth-graded multilayer structure, enabling the variation of the *C*_ff_ value within one BMG structure, the increase of resolving power would not be significant because of the small variation range in the *C*_ff_ value and, more importantly, the efficiency must be sacrificed for broader wavelength range (Yang *et al.*, 2016 ▸). Besides, we can always increase the line density of the BMG structure to achieve higher resolving power, but note that the multilayer structure distortion due to the sawtooth substrate would be more severe when the grating period is short (Voronov *et al.*, 2014 ▸), which reduces the actual efficiency, and the fabrication difficulty increases with grating line density.

## 4. Conclusion   

In summary, we have shown that the efficiency of cPGMs can be significantly improved by combining a multilayer-coated plane mirror (MM) and a blaze multilayer grating (BMG). The efficiencies of two combinations, MM+BMG- and SLM+BMG-based cPGMs, have been compared. The results show that coating a multilayer on both plane mirror and grating is preferable. Given the same resolving power and slit size, the advantage of multilayer-based cPGMs is that its higher efficiency gives higher flux compared with conventional single-layer cPGMs. The resolving power of a multilayer-based cPGM designed for ±1st order is usually lower than the maximum value that can be achieved by a conventional cPGM, because of the larger grazing-incidence angle and smaller *C*_ff_ value. This can be compensated by larger blaze angle, which is usually linked to high diffraction order, but this is restricted by the realistic values for the multilayer period and needs to be away from the TER region. Also, note that the larger blaze angle at high diffraction orders would add stricter requirements on fabrication precision and alignment.

Another important advantage of multilayer-based cPGMs is that high-order harmonics can be highly suppressed due to its refraction effects (Voronov *et al.*, 2016 ▸). In addition, both the higher diffraction order and lower *C*_ff_ value of BMG-based cPGMs lead to larger grazing-incidence angle for the plane mirror and gratings which is beneficial for reducing the beam footprint.

Alongside the fabrication feasibility of high-quality blazed multilayer gratings, this work now opens up the possibility to extend the energy range up to 4 keV by using high-efficiency multilayer-based cPGMs. Importantly, the proposed multilayer-based plane mirror and gratings are fully compatible with the current cPGM mechanics and can be potentially implemented as a future beamline upgrade.
